# Copy number gain of VCX, X-linked multi-copy gene, leads to cell proliferation and apoptosis during spermatogenesis

**DOI:** 10.18632/oncotarget.12397

**Published:** 2016-10-01

**Authors:** Juan Ji, Yufeng Qin, Rong Wang, Zhenyao Huang, Yan Zhang, Ran Zhou, Ling Song, Xiufeng Ling, Zhibin Hu, Dengshun Miao, Hongbing Shen, Yankai Xia, Xinru Wang, Chuncheng Lu

**Affiliations:** ^1^ State Key Laboratory of Reproductive Medicine, Institute of Toxicology, Nanjing Medical University, Nanjing, China; ^2^ Key Laboratory of Modern Toxicology of Ministry of Education, School of Public Health, Nanjing Medical University, Nanjing, China; ^3^ Epigenetics and Stem Cell Biology Laboratory, National Institute of Environmental Health Sciences, Research Triangle Park, NC, USA; ^4^ Department of Children Health Care, Nanjing Maternity and Child Health Care Hospital Affiliated to Nanjing Medical University, Nanjing, China; ^5^ Research Center for Bone and Stem Cells, Department of Anatomy, Histology, and Embryology, Nanjing Medical University, Nanjing, China; ^6^ Department of Epidemiology and Biostatistics and Key Laboratory of Modern Toxicology of Ministry of Education, School of Public Health, Nanjing Medical University, Nanjing, China

**Keywords:** copy number variations, non-obstructive azoospermia

## Abstract

Male factor infertility affects one-sixth of couples worldwide, and non-obstructive azoospermia (NOA) is one of the most severe forms. In recent years there has been increasing evidence to implicate the participation of X chromosome in the process of spermatogenesis. To uncover the roles of X-linked multi-copy genes in spermatogenesis, we performed systematic analysis of X-linked gene copy number variations (CNVs) and Y chromosome haplogrouping in 447 idiopathic NOA patients and 485 healthy controls. Interestingly, the frequency of individuals with abnormal level copy of Variable charge, X-linked (*VCX*) was significantly different between cases and controls after multiple test correction (*p* = 5.10 × 10^−5^). To discriminate the effect of gain/loss copies in these genes, we analyzed the frequency of X-linked multi-copy genes in subjects among subdivided groups. Our results demonstrated that individuals with increased copy numbers of Nuclear RNA export factor 2 (*NXF2*) (*p* = 9.21 × 10^−8^) and *VCX* (*p* = 1.97 × 10^−4^) conferred the risk of NOA. *In vitro* analysis demonstrated that increasing copy number of *VCX* could upregulate the gene expression and regulate cell proliferation and apoptosis. Our study establishes a robust association between the *VCX* CNVs and NOA risk.

## INTRODUCTION

Male infertility, a common reproductive disorder, is estimated to affect approximately one-sixth of couples worldwide [1]. A significant proportion of male infertility is accompanied by idiopathic azoospermia, most often presenting as non-obstructive azoospermia (NOA), defined as the absence of sperm in the ejaculate without the obstruction of reproductive tract. Certain pathogenic genetic causes, especially the Yq microdeletions, in male infertility are well established so far [2–4]. However, X-linked genes and their potential effects on male infertility are less well understood.

Originally, it was thought that mammalian X chromosomes were enriched in spermatogenesis genes expressed before meiosis and deficient after meiosis [5–7]. The paucity of post-meiotic genes on the X chromosome has been interpreted as a consequence of Meiotic Sex Chromosome Inactivation (MSCI) [8, 9]. Recently, Zhang et al. noticed that some testis-specific genes could be detected throughout all stages of spermatogenesis, indicating that they escape MSCI [10]. And it was estimated that 70% of these genes were expressed in post-meiotic stages [10]. Interestingly, the majority of these genes are multi-copy X-linked genes, moreover, the more copies of a gene, the higher chance to escape from X inactivation, accounting for higher expression level of multi-copy X-linked genes than that of single-copy X-linked genes [11].

With the rapid development of genome-scanning technologies [12], the structural variations in human genome were emerged and fully uncovered [13]. One type of structural variations is copy number variation (CNV) that generally defined as a segment of DNA (1 kb or larger), presenting at a variable copy number compared to a reference genome [13]. It has been demonstrated that CNV was associated with numerous diseases [14], including Alzheimer's disease, Parkinson disease, congenital scoliosis [15–17]. Recently, increasing evidence implied that CNVs were crucial determinant of spermatogenesis and might contribute to male infertility [18–20], while little is known about the relationship between X-linked gene CNVs and NOA.

To systematically analyze X-linked gene CNVs and their effects on NOA, we performed comprehensive molecular analyses in 447 idiopathic NOA men and 485 healthy controls. Eventually, the CNVs of Variable charge, X-linked (*VCX*) and Nuclear RNA export factor 2 (*NXF2*) were found to be associated with NOA, subsequently *in vitro* analysis was carried out to clarify the potential role of *VCX* in spermatogenesis.

## RESULTS

### Characteristics and clinical parameters of the study population

Our study included 447 NOA patients and 485 fertile controls. The mean age and BMI in control group were 30.1 and 24.3, and in case group was 29.5 and 23.3, respectively (Table 1). There were no significant differences identified between the case and control groups with regard to their age, smoking and drinking status. However, significant differences were identified in BMI between cases and controls (Table 1).

**Table 1:** Main characteristics and clinical parameters of study subjects

Variables	Controls (*n* = 485)	Cases (*n* = 447)
Age (years) [mean ± SD]	30.1 ± 4.7	29.5 ± 5.0
Smoking Status [*n*, (%)]		
Never Smokers	329 (68%)	318 (71%)
Ever Smokers	156 (32%)	129 (29%)
Alcohol consumption [*n*, (%)]		
Never drinkers	332 (68%)	313 (70%)
Ever drinkers	153 (32%)	134 (30%)
BMI (kg/m^2^) [mean ± SD]	24.3 ± 3.2	23.3 ± 3.0^a^

a *P* < 0.05 for Student's *t* test and Wilcoxon rank sum test for selected characteristics distributions between the control and case groups.

### Y-hg distribution between the case and control groups

To test for the potential influence of genetic backgrounds, 14 Y chromosome binary markers were used to define 14 Y-hgs in patients and normal subjects (controls). No significant difference in the Y-hg distribution was found between the healthy control group and the NOA case group (Supplementary Material, Table S1), which suggested that the genetic background, mainly Y-hgs, may not affect our results of the present association study.

### X-linked multi-copy gene copy number variations and NOA

Overall, seven X-linked gene copy numbers were determined in 447 NOA patients and 485 healthy controls using the AccuCopy method. The distributions of copy number of seven genes in case and control groups were shown in Table 2. We found that the frequency of individuals with abnormal copy number of *NXF2* (OR, 2.46, 95% CI 1.15^−^5.25, *p* = 1.66 × 10^−2^) and *VCX* (OR, 2.53, 95% CI 1.60^−^4.02, *p* = 5.10 × 10^−5^) in NOA group was significantly higher than that in control group, while the frequency of *FAM47* (OR, 0.27, 95% CI 0.07^−^0.95, *p* = 2.89 × 10^−2^) was significantly lower in NOA groups (Table 2). However, only the association between *VCX* and NOA risk retained after Bonferroni correction (Table 2).

**Table 2:** Distributions of *CSAG, CTAG, CT45, FAM47, H2AFB1, NXF2* and *VCX* gene copy numbers in subjects

Gene^a^	Fertile Controls (485)		Infertile Cases (447)		OR(95%CI)	*P* value
	Common level copies *n* (%)	Abnormal level copies *n* (%)	Common level copies *n* (%)	Abnormal level copies *n* (%)		
*CSAG*	470 (96.91)	15 (3.09)	423 (94.63)	24 (5.37)	1.78 (0.92−3.43)	8.29× 10^−2^
*CT45*	198 (40.82)	287 (59.18)	162 (36.24)	285 (63.76)	1.21 (0.93−1.58)	1.51× 10^−1^
*CTAG*	467 (96.29)	18 (3.71)	430 (96.20)	17 (3.80)	1.03 (0.52, 2.02)	9.41× 10^−2^
*FAM47*	473 (97.53)	12 (2.47)	444 (99.33)	3 (0.67)	0.27 (0.07, 0.95)	2.89× 10^−2^
*H2AFB1*	462 (95.26)	23 (4.74)	414 (92.62)	33 (7.38)	1.60 (0.93, 2.77)	9.02× 10^−2^
*NXF2*	475 (97.94)	10 (2.06)	425 (95.08)	22 (4.92)	2.46 (1.15−5.25)	1.66× 10^−2^
*VCX*	456 (94.02)	29 (5.98)	385 (86.13)	62 (13.87)	2.53 (1.60, 4.02)	5.10× 10^−5^^†^

† *P* value retained after multiple test correction.

To discriminate the effects of copy number gain or loss in these genes on NOA, we subdivided the subjects into three subgroups: the common level copy group, the less than common level group and the more than common level group. The detailed distributions were shown in Table 3. Our results demonstrated that 10 out of 485 (~2%) was found with decreased *NXF2* copy number in the control group, while no one was found in the NOA group. On the contrary, 22 out of 447 (~5%) were found with increased *NXF2* copy number in NOA group, while no one was found in the control group. Namely, decreased *NXF2* copy number showed protective against NOA (*p* = 2.20 × 10^−3^), while increased copy number conferred the risk of NOA (*p* = 9.21 × 10^−8^).

**Table 3:** The distribution of copy number variation of selected X chromosome multicopy genes in the azoospermia and normozoospermia groups

Gene	CNV	Azoospermia (447)		Normozoospermia (485)		Codominant Model	
		*n*	%	*n*	%	OR	*P* value
***CSAG***	Common level copy(4)	423	94.63	470	96.91	-	-
	Less than common level	3	0.67	0	0.00	-	1.07× 10^−2^
	More than common level	21	4.70	15	3.09	1.56 (0.79−3.06)	2.00× 10^−1^
***CT45***	Common level copy(6)	162	36.24	198	40.82	-	-
	Less than common level	156	34.90	144	29.69	1.32 (0.97−1.80)	7.30× 10^−2^
	More than common level	129	28.86	143	29.48	1.10 (0.80−1.51)	5.45× 10^−1^
***CTAG***	Common level copy(3)	430	96.20	467	96.29	-	-
	Less than common level	0	0.00	0	0.00	-	1.00
	More than common level	17	3.80	18	3.71	1.03 (0.52−2.02)	9.41× 10^−1^
***FAM47***	Common level copy(2)	444	99.33	473	97.53	-	-
	Less than common level	0	0.00	0	0.00	-	1.00
	More than common level	3	0.67	12	2.47	0.27 (0.75−0.95)	4.10× 10^−2^
***H2AFB1***	Common level copy(3)	414	92.62	462	95.26	-	-
	Less than common level	2	0.45	0	0.00	-	2.24× 10^−1^
	More than common level	31	6.94	23	4.74	1.50 (0.86−2.62)	1.50× 10^−1^
***NXF2***	Common level copy(4)	425	95.08	475	97.94	-	-
	Less than common level	0	0.00	10	2.06	-	2.20× 10^−3^
	More than common level	22	4.92	0	0.00	-	9.27× 10^−8^
***VCX***	Common level copy(4)	385	86.13	456	94.02	-	-
	Less than common level	6	1.34	2	0.41	3.55 (0.71−17.71)	1.22× 10^−1^
	More than common level	56	12.53	27	5.57	2.46 (1.52−3.97)	1.97 × 10^−4^

To the *VCX* gene, the frequency of individuals with increased *VCX* gene copy number in the NOA group (56 out of 447, ~13%) was significantly higher than that in the fertility/normozoospermia group (27 out of 485, ~6%) (OR, 2.46, 95% CI 1.52^−^3.97, *p* = 1.97 × 10^−4^).

### *VCX* expression in germ cells and seminal plasma

To explore the transfection efficiency of VCX in 293 and GC cell lines, we measured the VCX expression before and after transfection. As it showed (Supplementary Material, Figure S1A–S1C), the VCX expression was significantly increased after transfected. Besides, to investigate whether the VCX copy number gains lead to mRNA overexpression, the mRNA level of VCX in seminal plasma was detected, and we found that the mRNA expression of VCX was increased in NOA (Supplementary Figure S1D).

### Effects of *VCX* on cell proliferation, cell apoptosis and cell cycle

**Figure 1:**
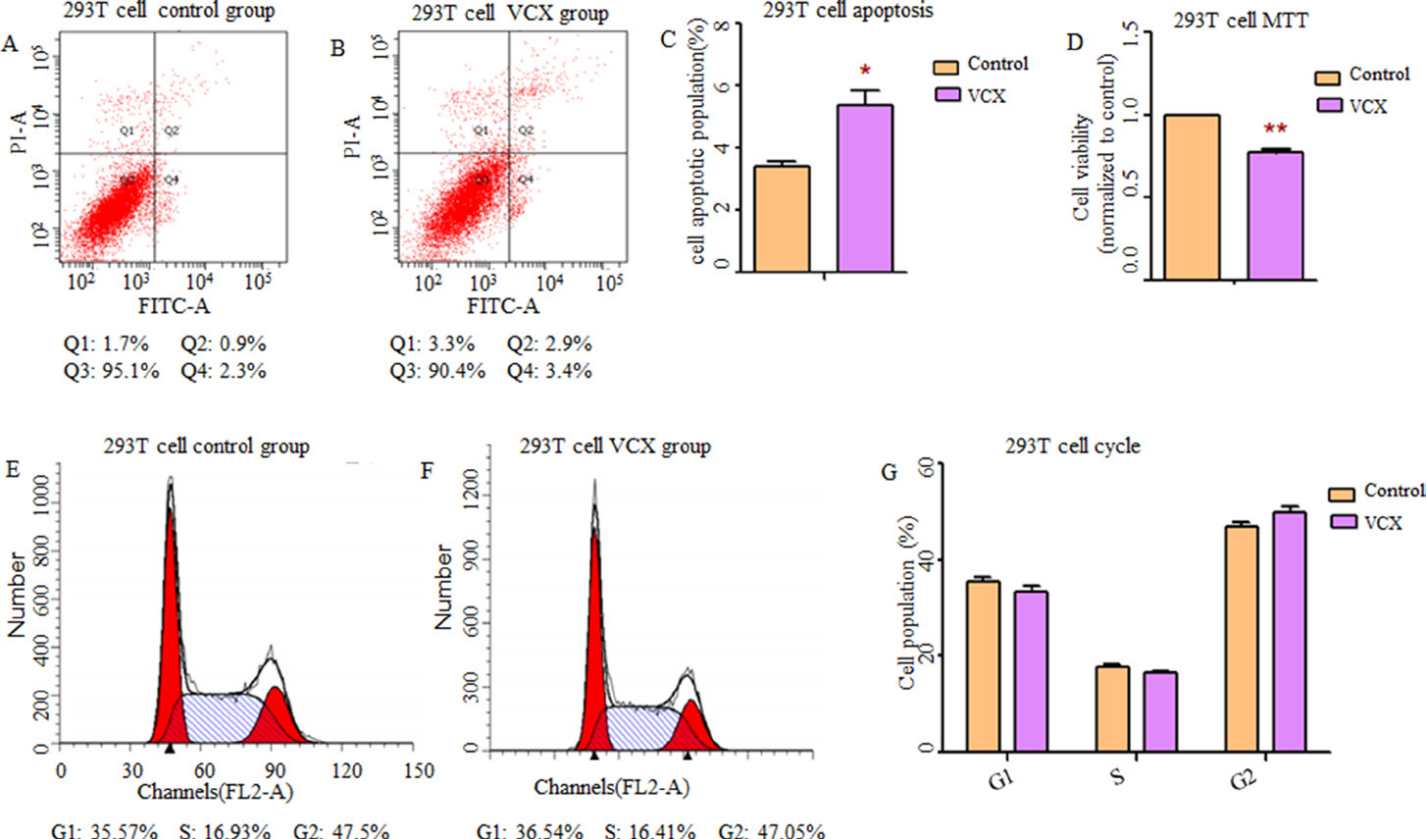
Effect of *VCX* on cell apoptosis, cell proliferation and cell cycle in 293T cells (**A**, **B**) Assessment of cell apoptosis was detected by flow cytometry. Cells in the Q2 and Q4 quadrant were late apoptotic and early apoptotic, respectively. (**C**) The percentage of apoptotic cells (Q2 + Q4) was presented in histogram and there was significant up-regulation of cell apoptosis in 293T cells transfected with *VCX*. (**D**) Cell growth activity was markedly inhibited. (**E**, **F**) representative histogram depicting cell-cycle profiles of control group and *VCX* group, respectively. (**G**) Various phases of the cell cycle was showed and there was no significant difference between the two groups, Each data point represented the mean ± SE from three separate experiments in which treatments were performed in triplicate. *P < 0.05.

**Figure 2:**
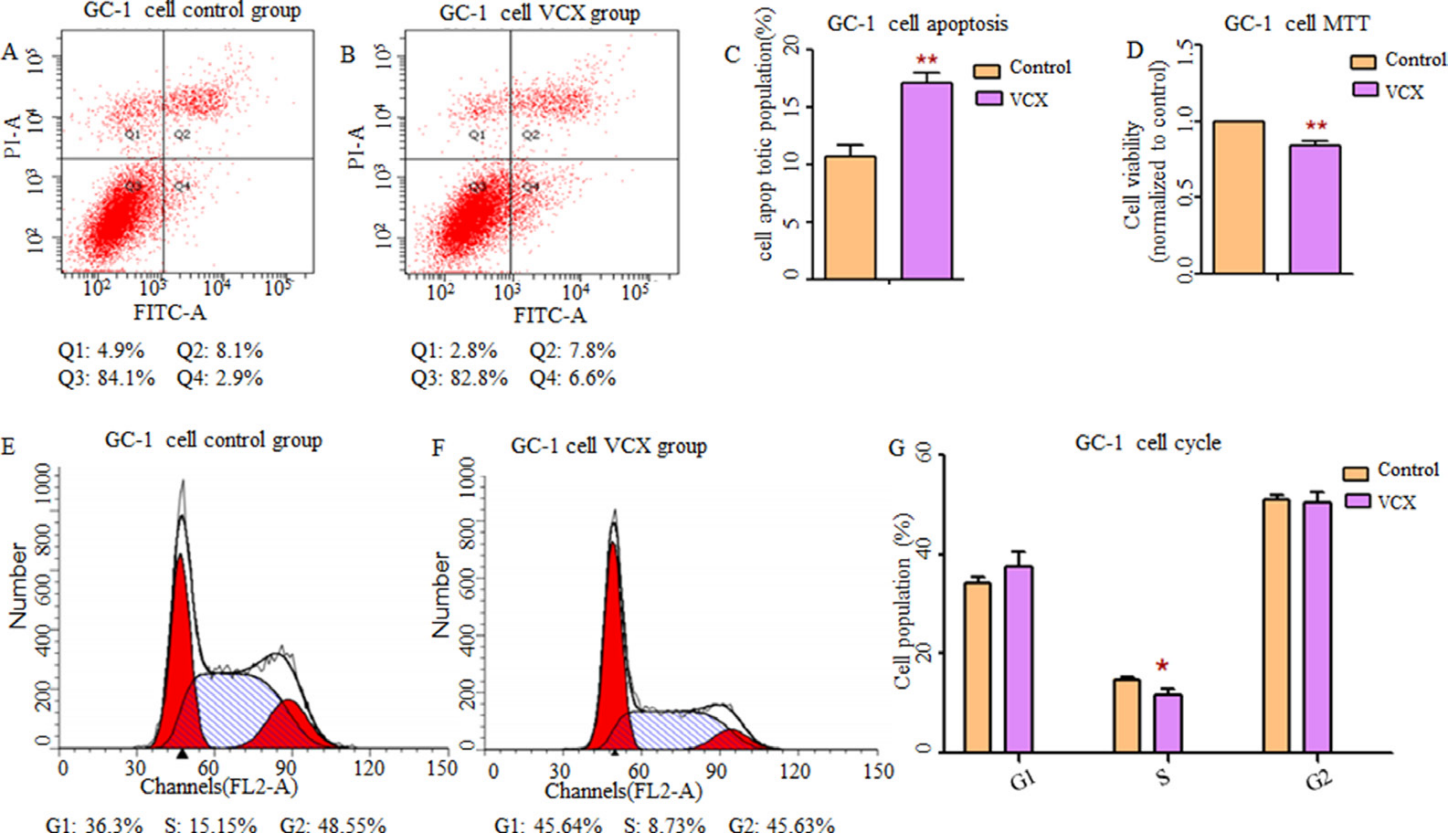
Effect of *VCX* on cell apoptosis, cell proliferation and cell cycle in GC-1 cells (**A**, **B**) Representative histogram depicting cell apoptosis profiles of indicated cells in control group and VCX group, respectively. (**C**) The percentage of apoptotic cells was increased significantly in GC-1 cells transfected with *VCX*. (**D**) Cell proliferation was markedly inhibited. (**E**, **F**) Data of the experiment were expressed as a percentage of total cells. Results quantitated in cell cycle were shown in (**G**) respectively. Each data point represented the mean ± SE from three separate experiments in which treatments were performed in triplicate. *P < 0.05, **P < 0.01.

**Figure 3:**
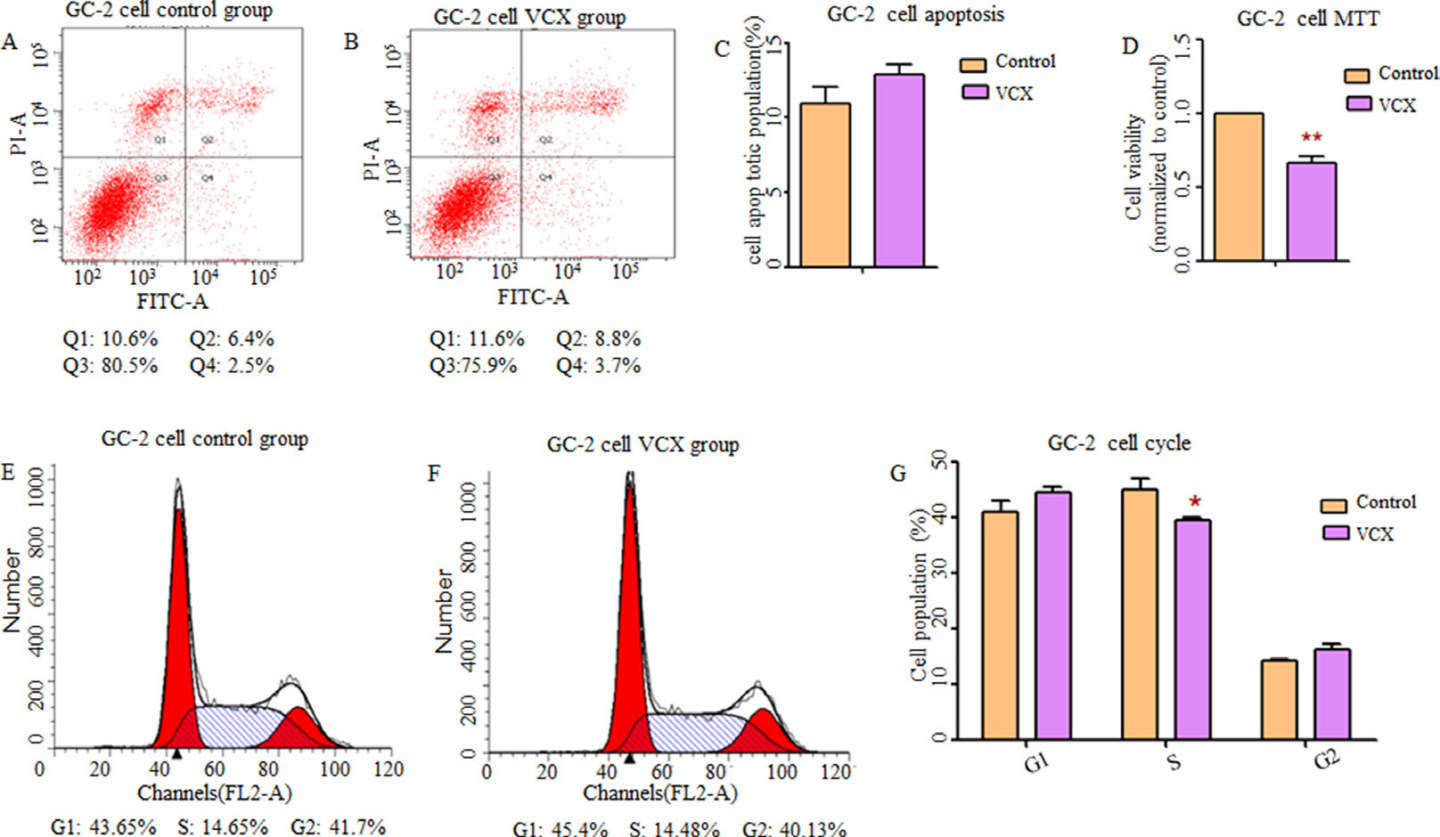
Effect of *VCX* on cell apoptosis, cell proliferation and cell cycle in GC-2 cells (**A**, **B**, **C**) There was no significant difference in cell apoptosis between control group and VCX group. (**D**) Cell proliferation was markedly down-regulated in VCX group. (**E**, **F**, **G**) Cell counts in S-phase of cell cycle were markedly reduced in VCX group. Each data point represented the mean ± SE from three separate experiments in which treatments were performed in triplicate. *P < 0.05.

By searching Database of Genomic Variants (DGV), we found that most frequent structure variations in *NXF2* were copy number loss, and didn't match what we observed that gaining copy was a risk in NOA. Additionally, its frequency was much lower (27/873, 3%) than *VCX* (2/9, 22%). Thus, in the functional study, we only included *VCX*. In an attempt to determine whether *VCX* have effects on cell functions, we over expressed *VCX* in 293T cells, GC-1 and GC-2 cells. As shown in Figure 1, cell apoptosis was significantly increased in the 293T cells (*p* = 1.76 × 10^−2^, Figure 1A–1C) and GC-1 cells (*p* = 9.40 × 10^−3^, Figure 2A–2C) after over expressed *VCX*. Although no significant difference of apoptosis was found in GC-2 cells, similar trends were observed (Figure 3A–3C), suggesting that VCX exerts a growth inhibitor. To further validate this, we carried out MTT (3-(4,5-dimethyl-2-thiazolyl)-2,5-diphenyl-2-H-tetrazolium bromide) assays. Consistent with the cell apoptosis analysis, cell proliferation significantly decreased in cells with high expression level of *VCX* (*p* = 9.47 × 10^−3^, Figure 1D; *p* = 9.99 × 10^−3^, Figure. 2D; *p* =1.70 × 10^−3^, Figure 3D), revealing that growth inhibition was accompanied with increased apoptosis. Besides, high expression level of *VCX* affected cell cycle in GC-1 cells (*p* = 4.35 × 10^−2^, Figure 2E–2G) and GC-2 cells (*p* = 3.69 × 10^−2^, Figure 3E–3G), indicating that *VCX* delayed cell progression in G1 to S transition. However, no significant difference was observed in 293T cells (Figure 1E–1G).

## DISCUSSION

X chromosome was derived from autosomal chromosome and under natural selection, and its gene content changed largely, some of which were multi-copy genes [21]. The roles of X-linked CNVs in cognitive disorders [22, 23], primary ovarian insufficiency [24] and premature ovarian failure [25] have been investigated extensively, while few studies have reported the associations between X-linked CNVs and male infertility. Recently, accumulating evidence revealed that infertile men had significantly higher duplication or deletion load than normal controls [26–28].

In this study, we hypothesize that the copy number variations of X-linked multi-copy genes confer the risk of male infertility. We explored the role of X-linked CNVs in 447 individuals with NOA and 485 healthy controls by applying AccuCopy assay. Amazingly, we found that the gaining more copies of *VCX* and *NXF2* were associated with NOA.

*NXF2*, encoded by multigene families on X chromosome, is a germ cell-specific gene [29]. It has been demonstrated that *NXF2* play functions in male meiosis, spermatogonial proliferation as well as maintenance of spermatogonial stem cells [30, 31]. And the deletion of *NXF2-NXF3* genomic region on the X chromosome in male germline causes male infertility in mice [32]. Contrary to previous studies, our results demonstrated that the copy number increase of *NXF2* conferred the risk of NOA. Functional studies, including both the gain and loss of this gene, are needed to explore the potential mechanisms in the future.

*VCX* localizes on cytogenetic band Xp22.3 and contains 4 copies [33]. From DGV, we found most structure variations in Xp23 are gaining the gene copies (including *VCX*). Thus, we overexpressed *VCX in vitro* for the further investigation. As one member of novel cancer/testis (CT) antigen [34], *VCX* exclusively expresses in testis and is most likely restricted to male germ cells [35], moreover, its expression is regulated epigenetically [36]. It has been demonstrated that *VCX* was overexpressed in a subset of non-small cell lung cancer (NSCLC) cell lines and tumor tissues [37, 38], suggesting that it has oncogenic role [34]. To explore the role of *VCX* in spermatogenesis, functional analysis was performed in 293T, GC-1 and GC-2 cell lines, and our results demonstrated that *VCX* promoted cell apoptosis and inhibited cell growth, contributing to spermatogenesis impairment [39], which was probably by mediating mitochondria-dependent apoptosis pathway [40] or p53-Bax pathway [41]. Moreover, *VCX* delayed cell-progression in G1 to S transition, resulting in cell division disorder and spermatogenic failure.

In conclusion, our findings revealed that copy number gain of *VCX* attributed to NOA by an underlying mechanism that induced cell apoptosis, inhibited cell proliferation and disrupted cell cycle progression, thus *VCX* may be a potential biomarker for the susceptibility to NOA.

## MATERIALS AND METHODS

### Study population and sample collection

Study subjects were volunteers from the affiliated hospitals of Nanjing Medical University between 2010 and 2014 (NMU Infertile Study) and described previously [42, 43]. All infertile male subjects were Han Chinese men determined to have idiopathic NOA and selected on the basis of comprehensive andrological testing, including examination of medical history, physical examination, semen analysis, scrotal ultrasound, hormone analysis, karyotyping and Y chromosome microdeletion screening. Those with a history of cryptorchidism, vascular trauma, orchitis, obstruction of the vas deferens, vasectomy, abnormalities in chromosome number or microdeletions of the azoospermia factor region on the Y chromosome were excluded from the study. Subjects with NOA had no detectable sperm in the ejaculate after evaluation of the centrifuged pellet. To ensure the reliability of the diagnosis, each individual was examined twice, and the absence of spermatozoa from both replicate samples was taken to indicate azoospermia. The controls had fathered one or more healthy child and were frequency matched to the cases on the basis of age and area of residence. The protocol and consent form were approved by the Institutional Review Boards of Nanjing Medical University. All activities involving human subjects were done under full compliance with government policies and the Helsinki Declaration. Totally, 447 patients with non-obstructive azoospermia and 485 matched healthy male controls were recruited in this study. Written informed consents were obtained from all of them. After completing a questionnaire with detailed information, each subject donated 5 ml of blood for genomic DNA extraction.

### Y chromosome haplogrouping

14 highly informative polymorphic loci for East Asians (M130, YAP, M89, M9, M231, M120, M119, M268, M95, M176, M175, M122, M134 and M117) [44, 45] were used to define 14 Y-hgs following the nomenclature recommended by the Y Chromosome Consortium[46] and its update described by Sengupta et al [47].

The SNaPshot (Applied Biosystems, Foster City, CA) minisequencing reaction assay was used for polymorphism genotyping [48] The detailed experimental procedures have been well described in our previous study [49].

### CNV genotyping using the AccuCopy method

The AccuCopy technique, a CNV genotyping method based on multiplex fluorescence competitive amplification, was recently developed by Genesky Biotechnologies (Shanghai, China), and well described by Du et al [50]. Limited by the highly homologous sequences and the detect threshold of AccuCopy, only seven gene families were recruited in our study. In order to ensure the reliability of our results, we chose three target genomic segments within the CNV region for the *CT45* and *FAM47* genes, and two for the *NXF2*, *CSAG*, *CTAG*, *H2AFB1*, and *VCX*. According to the reaction condition and stability, these target segments were divided into two panels. The reference genome sequences were obtained from the UCSC Genome Browser (http://genome.ucsc.edu/ genome assembly hg19). Additionally, three reference segments used for normalization were screened and chosen at three loci of *2P*, *16P* and *20q*. The forward (F) and reverse (R) primers of these segments and the size of PCR product amplified from human genomic DNA are provided in Supplementary Table S2, respectively.

AccuCopy assay was modified as one multiplex competitive PCR amplification followed by one labeling extension, in which one of PCR primers for each fragment was synthesized with addition of a universal sequence, i.e. 5**′**ACACGACCGGTAACGCTTAGA3′ at 5′ end so that the PCR products can be FAM-labeled in a subsequent extension reaction using a 5′ FAM-modified primer i.e. FAMEF: 5′ [FAM] ACACGACCGGTAACGCTTAGA3′. All primers mentioned above were synthesized at Sangon Biotech (Shanghai, China). The competitive DNAs for the three reference and sixteen target segments were designed and synthesized in double strand by Genesky Biotechnologies and provided in a 200 × mixture. The sequences of synthesized competitive DNAs were almost the same as their reference homologies in the human reference genome except 2 bp deletion introduced. The detailed experimental procedures were described previously with minor modifications.

### Statistical analysis

Statistical analyses were carried out using Stata 10.0 statistical software package (Stata Corp, LP). Pearson's chi-squared test was used to demonstrate differences of categorical variables such as drinking and smoking status between cases and controls. Student's *t*-test was used to show differences in continuous variables such as age and body mass index (BMI). Associations between CNVs and the risk of NOA were evaluated by computing odds ratios (ORs) and their 95% confidence intervals (CIs) from logistic regression under different models. The Bonferroni adjustment for multiple testing was applied. The *P*-value for a truly significant result was calculated as 7.14 × 10^−3^ (0.05/7).

### Functional analysis of *VCX* CNV

By searching the DGV, we found that there are seven structure variations in *VCX* and most of them were copy number gains (Supplementary Material, Figure S2). Human VCX cDNA sequence (NM 013452.2) was synthesis by Realgene biotechnology company and inserted in expression vector pcDNA3.1 (+). The recombinant plasmid was confirmed by endonucleases digesting and DNA sequencing.

Three commonly used cell lines (293T cells, GC-1 cells and GC-2 cells) were employed for the function analysis *in vitro* [51–54]. All cell lines were cultured in DMEM (Gibco, USA), with 10% fetal bovine serum (FBS) (Gibco, USA), 100 U mL −1 penicillin (Gibco, USA), and 100 μgmL-1 streptomycin (Gibco, USA) at 37°C under 5% CO_2_. Cells were transfected with 4 μg of pcDNA-*VCX* or pcDNA-NC using Lipofectamine 2000 (Invitrogen, USA). After 24 h transfection, cells were stained with propidium iodide (PI) and annexin V for 30 min, then analyzed by FACS (BD Biosciences, USA) to quantify the cell apoptosis or cell cycle. Cell proliferation was analyzed 24 h after transfection by MTT Assay Kit (Beyotime, China). Each experiment was performed in triplicated independently.

## SUPPLEMENTARY MATERIALS


