# Supplementary material for: Copy number gain of VCX, X-linked multi-copy gene, leads to cell proliferation and apoptosis during spermatogenesis
Source: Oncotarget. 2016 Oct 1;7(48):78532–40. doi: 10.18632/oncotarget.12397 (PMC5340235; doi:10.18632/oncotarget.12397)
Supplement: Supplementary file 1 [file oncotarget-07-78532-s001.pdf]

Copy number gain of VCX, X-linked multi-copy gene, leads to cell proliferation and apoptosis during spermatogenesis

Supplementary Materials

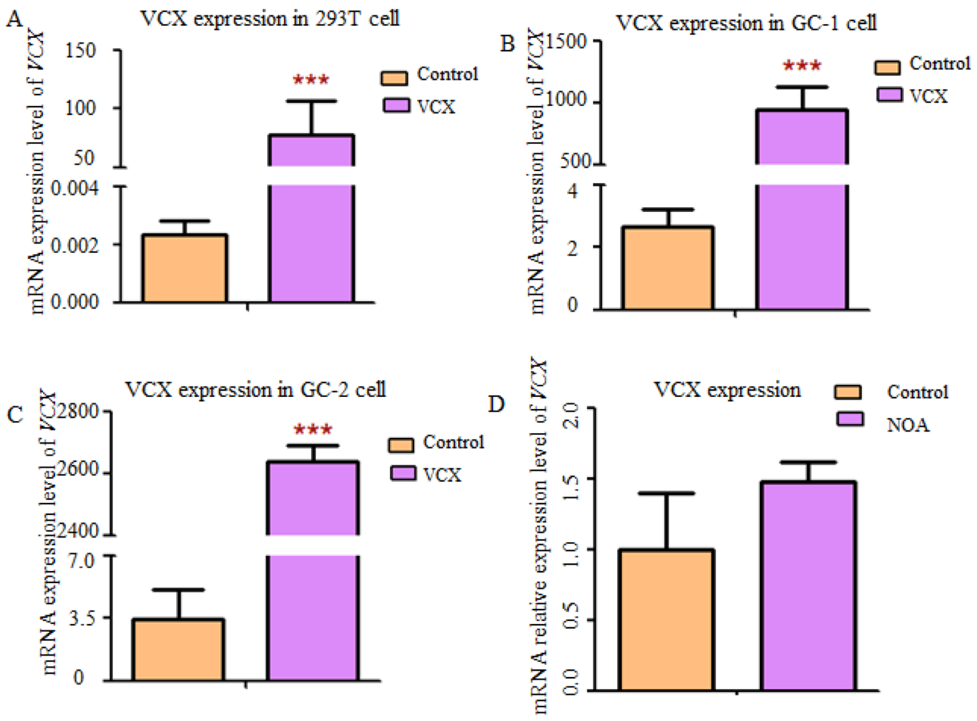

Supplementary Figure S1: *VCX* expression in cells and seminal plasma. (**A–C**) Expression level of VCX in 293T, GC-1 and GC-2 cells. (**D**) Expression level of VCX in seminal plasma in Controls (n = 3) and NOA patients (n = 3).

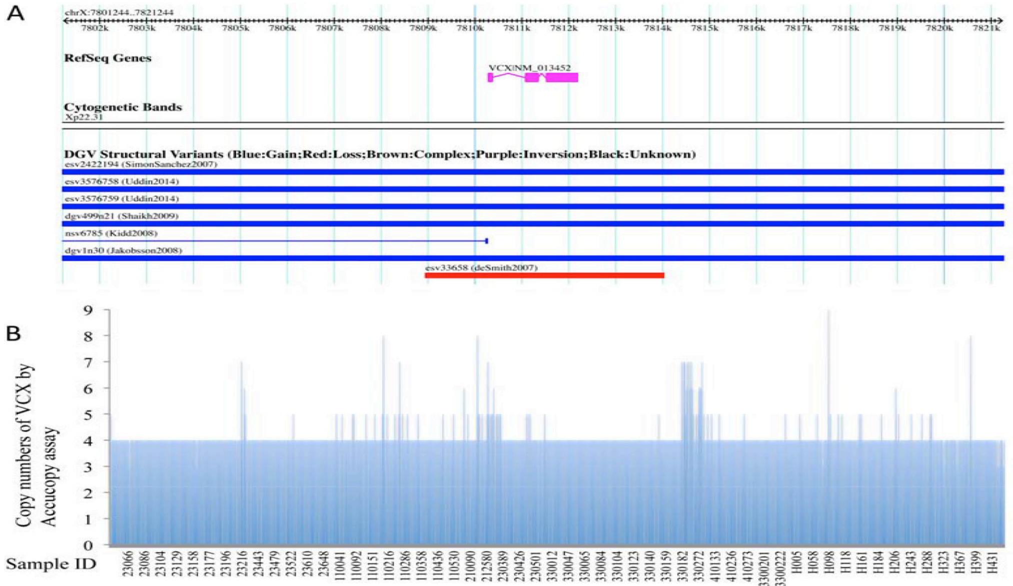

Supplementary Figure S2: The chromosome location of VCX with CNVs information and copy numbers of VCX in the study. (**A**) Schematic of *VCX* in Chr. Xp22 and the identified CNVs. (**B**) Copy number of *VCX* decteted by the Accucopy assay with a patented Multiplex AccuCopyTM Kit (Genesky Bio-Tech Co., Ltd., Shanghai, China).

Supplementary Table S1: Y haplogroup distributions in a healthy control Han Chinese population and the case population with non-obstructive azoospermia

Y-hgs	Normozoospermia (485)		Azoospermia (447)		OR	*P* value†
	n	%	*n*	%		
M130_C	48	9.90	43	9.62	0.97 (0.63−1.49)	0.8867
YAP_DE	4	0.82	3	0.67	0.81 (0.18−3.65)	1.0000
M89_F*	3	0.62	2	0.45	0.72 (0.12−4.34)	1.0000
M9_K*	1	0.21	4	0.89	4.37 (0.49−39.25)	0.2002
M231_N*	38	7.84	42	9.40	1.22 (0.77−1.93)	0.3954
M120_Q1	14	2.89	13	2.91	1.01 (0.47−2.17)	0.9842
M119_O1	59	12.16	60	13.42	1.12 (0.76−1.64)	0.5654
M95_O2a	15	3.09	15	3.36	1.09 (0.53−2.25)	0.8203
M268_O2*	25	5.15	22	4.92	0.95 (0.53−1.71)	0.8710
M176_O2b	1	0.21	1	0.22	1.09 (0.07−17.40)	1.0000
M175_O*	0	0	2	0.45	−	0.2298
M122_O3*	134	27.63	121	27.07	0.97 (0.73−1.30)	0.8482
M134_O3e*	71	14.64	53	11.86	0.78 (0.54−1.15)	0.211
M117_O3e1	72	14.84	66	14.77	0.99 (0.69−1.43)	0.9725

†Fisher's exact test.

Supplementary Table S2: The forward (F) and reverse (R) primers for multiplex competitive amplification

Gene	Forward Primer (F)	Reverse Primers(R)	Amplicon size (bp)
**Primers of nine target segments:**			
**NXF2-1**	**ACACGACCGGTAACGCTTAGA** GCCCTCCTTCTCCAGTTTCCTC	**GTTTCTT**GAGGTCTGTG GTCCTCCCAGAG	296
**NXF2-2**	**GTTTCTT**CTGGAGATCGA CAGGGTCTCCT	**ACACGACCGGTAACGCTTAGA**TTTTA AGAGGAGCAGGAGTGGGTGGT	230
**CT45-1**	**ACACGACCGGTAACGCTTAGA**TTT TAAACCCTGGTCCTCACTCACAGT TATG	**GTTTCTT**CAATGCGCACCTCAGAC TATGG	159
**CT45-2**	**GTTTCTT**GCTGAAGTCTCCTG GCATATGTTACC	**ACACGACCGGTAACGCTTAGA**AC CAGCACAGGTGGGAATACAAAAT	177
**CT45-3**	**GTTTCTT**TTGGTTGTATACACATATG CAGATTGC	**ACACGACCGGTAACGCTTAGA**CCAA CAGGGCCATCCTCTG	232
**CSAG-1**	**GTTTCTT**GGTCTGGTGAAGATGTC CAGGAA	**ACACGACCGGTAACGCTTAGA**C CAGGGGAGGACAGGGTAGG	199
**CSAG-2**	**GTTTCTT**TCAAGGAAGTTCCAGGAA CAAAAGG	**ACACGACCGGTAACGCTTAGA**TTTT TAGTGTTGGCCCACTCCATTCC	115
**CTAG-1**	**ACACGACCGGTAACGCTTAGA**GA GGGAGCCCAGGGAGATG	**GTTTCTT**GTCTGCAGCAGTCAGTCG GCTA	116
**CTAG-2**	**GTTTCTT**CCCTTCCTAGGTCAT GCCTCCT	**ACACGACCGGTAACGCTTAGA**ACAT GTAAGCCGTCCTCCTCCA	134
**H2AFB1-1**	**ACACGACCGGTAACGCTTAGA**CTG GACGCGGTAACTGTCCTCT	**GTTTCTT**CACCTGGCTCACTGAAAAC GAA	210

**H2AFB1-2**	**ACACGACCGGTAACGCTTAGA**CT GAGCACCCTTTTCAACACGA	**GTTTCTT**GGTGGACGAGTGGACCT GTCA	131
**FAM47-1**	**ACACGACCGGTAACGCTTAGA**TGT GACAAACCGCCTTCCAAGTA	**GTTTCTT**GCTTTCTTGAG CAGCTTTTTCTTCCT	267
**FAM47-2**	**ACACGACCGGTAACGCTTAG A**TTTTGAGTGTGTTTCT GACTCTCTTCAACGT	**GTTTCTT**TCCTAGGTCTC CAGCCCACTTG	110
**FAM47-3**	**ACACGACCGGTAACGCTTAGA**GAT GAACCCGACATTCTTGACGG	**GTTTCTT**GATGCATCTGTGAC GTCTTCTTTG	222
**VCX-1**	**GTTTCTT**TGCCACGTGATCTGC-CAAAAT	**ACACGACCGGTAACGCTTAGA**TTT TATGAGGAAGGCAGGGTGCTCTAA	159
**VCX-2**	**ACACGACCGGTAACGCTTAGA**CA CAGCTCAGGGGCGTGATT	**GTTTCTT**GAGGAG-GACTTCCTCTTTCCTGC	209
**Primers of four reference segments (*2P, 16P* and *20q*):**			
**2P**	**GTTTCTT**TGAGC CAAAAATTCAGAATACAAGGA	**ACACGACCGGTAACGCTTAGA**TT GCTTGGAAGGCAGGCAAAC	103
**16P**	**ACACGACCGGTAACGCTTAG A**TCCTCCACCAAGCTGATGTGTT	**GTTTCTT**TTCAGGCCTGTCCCC GAAATAG	326
**20q**	**ACACGACCGGTAACGCT TAGA**AGGGTGCTGGGATCAGAGA GAG	**GTTTCTT**GCTACTGGAGGGTG GCAAAATG	250
